# Best supportive care for patients with primary progressive multiple sclerosis (PPMS) in Germany prior to ocrelizumab treatment: Final results from the RETRO PPMS study

**DOI:** 10.1177/11795735241296001

**Published:** 2024-10-23

**Authors:** Herbert Schreiber, Iris-Katharina Penner, Tanja Maier, Stefanie Hieke-Schulz, Jost Leemhuis, Tjalf Ziemssen

**Affiliations:** 1 Neurological Practice Center, Neuropoint Academy & NTD, Ulm, Germany; 2 Department of Neurology, 27252 Inselspital, Bern University Hospital, University of Bern, Bern, Switzerland; 3 COGITO Center for Applied Neurocognition and Neuropsychological Research, Düsseldorf, Germany; 4 123188 Roche Pharma AG, Grenzach-Wyhlen, Germany; 5 Center of Clinical Neuroscience, 39063 Neurological University Clinic Carl Gustav Carus, Technical University of Dresden, Dresden, Germany

**Keywords:** Primary progressive multiple sclerosis, best supportive care, symptomatic therapy, off-label treatment, real-world data, retrospective

## Abstract

**Background:**

Best supportive care (BSC) measures are an essential component for the management of primary progressive multiple sclerosis (PPMS).

**Objectives:**

RETRO PPMS (ML39631) is the first study to systematically analyze the therapeutic journey and standard of BSC of patients with PPMS in Germany.

**Design:**

This multicenter, non-interventional study retrospectively analyzed patient charts. Methods: Data were recorded up until the first infusion of ocrelizumab (July 2018 to October 2021). Medical history, disease status, disease activity and treatments were assessed from 12 months before PPMS diagnosis until study start. Acute interventions, BSC parameters and rehabilitation measures from the past 27 months were assessed.

**Results:**

The core analysis population (N = 462) had a mean age (range) of 57.4 (27–85) years and mean disease duration of 13.7 (0.3–55.2) years. The most frequently reported symptoms were muscle spasticity, bladder disorder, ataxia, gait disturbance and fatigue. The most commonly used treatment was physical/occupational therapy (66.5% of patients); 47.2% received off-label treatment with corticosteroids/disease-modifying therapies. BSC measures for many symptoms were strikingly rare – especially for fatigue and cognitive impairment.

**Conclusion:**

This analysis uncovers severe BSC deficits for many debilitating PPMS symptoms. There is still a large unmet need for innovative multidisciplinary care concepts and improvements in neurological primary and secondary care.

## Introduction

Primary progressive multiple sclerosis (PPMS) affects ∼10% of the 280 000 patients with MS in Germany.^1^ With this condition, disability accumulates continuously and at a faster rate than in relapsing-remitting MS (RRMS): the median time to reach an expanded disability status scale (EDSS) of 6.0 is 14 years.^2^

PPMS affects all functional systems; common symptoms include mobility decline, spasticity, cognitive impairment, bladder dysfunction, fatigue, ataxia, pain and depression.^3^ Best supportive care (BSC) measures complement disease-modifying treatments (DMTs) and are an essential component of MS management to improve patients’ quality of life. Symptomatic BSC therapies aim to maintain functional capabilities for as long as possible, to prevent secondary complications and to reduce the social and emotional burden of the disease. They include pharmacological treatments and a wide spectrum of non-pharmacological treatments such as physiotherapy, occupational and speech therapy, psychological therapy/support, the provision of devices such as walking aids and wheelchairs, rehabilitation programs, as well as acute, palliative and nursing care measures.

Although increased awareness and improvements in symptomatic treatment options have led to more frequent BSC interventions for most MS symptoms, data from the German MS registry suggest that common and disabling symptoms nevertheless remain untreated in many patients with PPMS.^4^ These registry data may be limited by variable quality and lack of a standardized methodological approach, as systematic data on BSC practices among the German PPMS population are scarce.

RETRO PPMS (ML39631) is the first study aiming to systematically assess the therapeutic journey and the real-world standard of care in clinical practice for patients with PPMS in Germany since 2018, prior to the availability of the first approved DMT, ocrelizumab (Ocrevus^®^, F. Hoffmann-La Roche AG, Basel, Switzerland).^5^ BSC measures were retrospectively analyzed based on patient chart reviews.

## Methods

### Study design

RETRO PPMS is a multicenter, secondary-data-use study of patients with PPMS in Germany to document BSC treatment in clinical practice. Due to the non-interventional and retrospective design, any treatment decisions were taken prior to and without influence of study participation.

Retrospective data collection by patient chart review started on July 19, 2018; the database was closed in October 2021. Eligible patients were ≥18 years old, had a confirmed PPMS diagnosis according to the revised McDonald 2010 criteria.^6^ Patients who started treatment by physician’s choice as well as patients without therapy were included. If patients were being treated with ocrelizumab, data were extracted only until they received the first dose. All eligible patients visiting the participating physicians after study start were included consecutively. The physicians assessed medical records to determine patient eligibility and completed electronic case report forms (eCRF) for the clinical database.

### Endpoints

The primary endpoint was the spectrum and frequency of different BSC measures administered to patients with PPMS in daily routine for integral symptom management. BSC was defined as individualized supportive care measures, including medications, to manage and alleviate PPMS symptoms.

BSC measures for the following symptoms from 2 years prior to study start were recorded: fatigue, bladder dysfunction, spasticity, ataxia, tremor, sexual/erectile dysfunction, depression, pain, defecation disorder, cognitive impairment and “other”. The latter were analyzed as a separate category to avoid bias, although they were subsequently coded using the Medical Dictionary for Regulatory Activities (MedDRA). Each symptom was analyzed individually, differentiating between pharmacological and non-pharmacological (physical/occupational therapy, psychotherapy, alternative healing methods) treatments.

Secondary endpoints were off-label treatment with DMTs or corticosteroids, disease status by change in EDSS, Multiple Sclerosis Severity Score (MSSS) and Clinical Global Impression (CGI) score from baseline (12 months prior to PPMS diagnosis), frequency of comorbidities, including psychiatric disorders (12 months prior to diagnosis), frequency of hospitalizations/emergency visits/rehabilitation measures/devices and aids (2 years prior to study start), change in disease activity as measured by MRI lesions and adverse events (AEs) (12 months prior to diagnosis, respectively).

### Statistics

The initial target sample size of 1070 patients at 180 centers was reduced to ≥400 patients from ≤80 centers, which represents a sufficiently large sample to analyze the relative frequency of any BSC therapy using a 95% confidence interval with the envisaged precision of ≥4–5% (assuming a worst case with 50% incidence). All analyses were based on the core analysis population (CAP), which comprised all enrolled patients who met the eligibility criteria.

A descriptive analysis of BSC measures was performed in the overall population and for subpopulations by PPMS symptom. Absolute and relative frequencies with 95% confidence intervals were derived by the Clopper-Pearson method. Subgroup analyses were based on the last EDSS score (<4 vs ≥4). According to the concept of MS as a two-stage disability progression,^7^ this threshold enables differentiation of early-phase patients from those who have already entered a stage dominated by neurodegenerative decline, with a relatively uniform disability progression. Moreover, the parameters time since diagnosis (>10 years vs ≤10 years) and gender were assessed.

Exploratory secondary outcome measures were analyzed descriptively. EDSS values were summarized by visits including changes from baseline, which referred to the 12-month interval prior to PPMS diagnosis. Total EDSS scores were either automatically determined within the eCRF according to the Neurostatus EDSS scale^8^ using individual, discrete functional system score entries or – if those were not available – EDSS scores were entered directly by the investigator. At defined post-baseline time intervals, average values and changes from baseline were analyzed. The analysis of continuous data included the number of patients with non-missing values, mean, standard deviation (SD), median, range (min, max) and interquartile range (Q1, Q3). Categorical data was analyzed using absolute and relative frequencies (percentages of all non-missing values). All available data were included in the analyses and summarized if possible; missing data were generally not substituted.

## Results

### CAP

At recruitment termination, 463 patients from 53 centers were enrolled. One patient did not have a confirmed diagnosis of PPMS according to the McDonald 2010 criteria, therefore the final CAP included 462 patients.

Baseline characteristics of the CAP are shown in Table 1. On average, 4 years passed between the onset of the first PPMS symptoms and the established diagnosis of PPMS.

**Table 1.** Baseline demographics and disease characteristics.

	Core Analysis Population (N = 462)
Age^a^, years	57.4 (27–85)
Female sex, n (%)	258 (55.8)
Years from first MS symptoms to study start^a^	13.7 (0.3–55.2)
Years from PPMS diagnosis to study start^a^	9.4 (0.0–51.8)
EDSS score^a,b^, n = 53	3.83 (1.0–8.0)
Number of patients with >1 MRI lesion^b^	
>1 T1 lesions, n = 120	20 (16.7%; 76.7% unknown status)
>1 T2 lesions, n = 120	70 (58.3%; 38.3% unknown status)
>1 Gd lesions, n = 25	13 (52.0%; 36.0% unknown status)

^a^Mean (range).

^b^12 months prior to MS diagnosis. EDSS and MRI data were only available for a subset of patients. EDSS, Expanded Disability Status Scale; Gd, Gadolinium; MRI, magnetic resonance imaging; MS, multiple sclerosis; PPMS, primary progressive MS.

### PPMS symptoms

**Figure 1.**
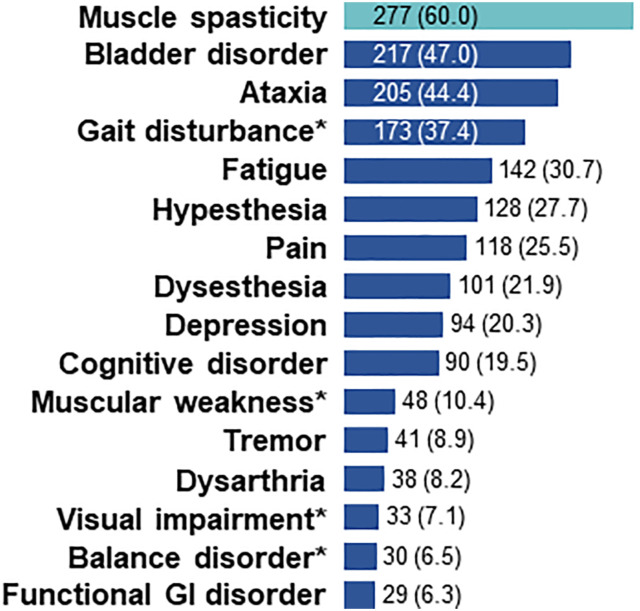
Ranking of overall PPMS symptoms. Overall PPMS symptoms in the period of 12 months prior to PPMS diagnosis with incidence >5% are presented; *symptoms reported in the category “others”. Percentages are based on the total number of patients (N = 462). GI, gastrointestinal; PPMS, primary progressive multiple sclerosis.

The most frequent initial PPMS symptoms were muscle spasticity (24.0% of patients) and ataxia (22.1%), followed by gait disturbances (21.2%). This triad of symptoms, representing a complex gait disorder, also ranked among the most common overall PPMS symptoms (Figure 1); where bladder disorder ranked as the second most frequent overall symptom. Notably, fatigue and cognitive impairment were only reported in approximately one third and one fifth of patients, respectively.

### Non-pharmacological and pharmacological BSC

Overall, 72.9% of patients received non-pharmacological symptomatic therapies (66.5% physical/occupational therapy, 4.8% psychotherapy and 1.7% alternative healing methods). In particular, physical/occupational therapy was offered to 74.0% of patients with an EDSS ≥4 (n = 258), compared with 52.1% with an EDSS <4 (n = 71), and to more patients with >10 years since PPMS diagnosis (77.4% of n = 159) than to patients with a shorter disease duration (60.7% of n = 303).

The most commonly used symptomatic pharmacological therapies were fampridine (26.0% of patients) and baclofen (21.0%), followed by nabiximols (cannabidiol/tetrahydrocannabinol, THC; 8.2%), pregabalin (6.3%), citalopram (5.0%), tizanidine (5.0%) and gabapentin (4.3%). When compared with patients having an EDSS <4, considerably greater percentages of patients with higher EDSS scores received fampridine (34.1% vs 16.9%), baclofen (29.1% vs 11.3%), cannabidiol/THC (10.9% vs 2.8%) and pregabalin (8.1% vs 2.8%). More patients with >10 years since diagnosis were treated with baclofen (29.6% vs 16.5%) and cannabidiol/THC (11.9% vs 6.3%) than patients with ≤10 years.

### BSC analyzed by symptom

**Figure 2.**
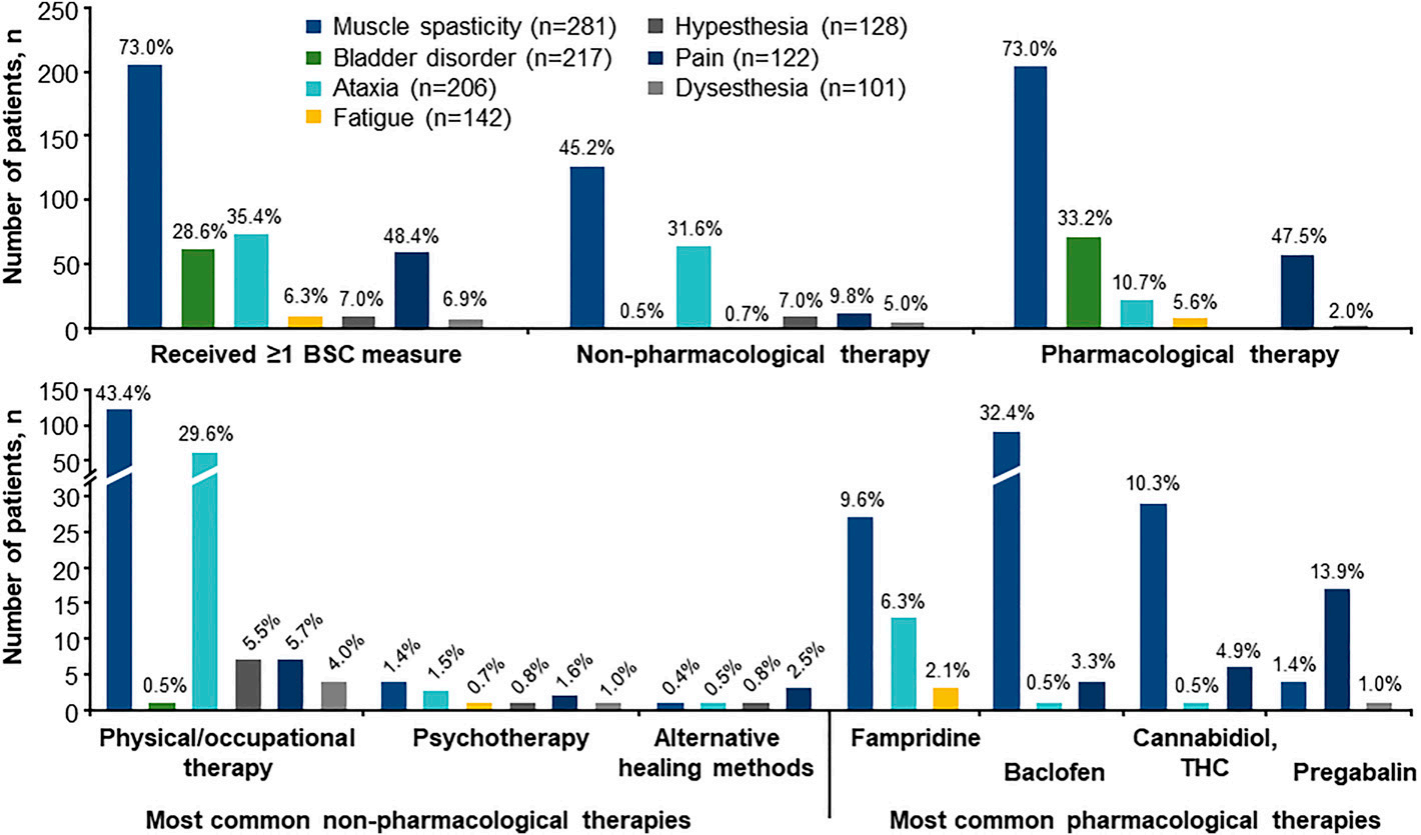
BSC measures related to the seven most common symptoms. BSC measures received within 27 months prior to study start are included, presented by symptom with n > 100, except symptoms reported in the category “others”. Percentages are based on the number of patients presenting with the respective symptom (total population: N = 462). BSC, best supportive care; THC, tetrahydrocannabinol.

While more than two thirds of patients with muscle spasticity and nearly half of patients with pain received ≥1 BSC measure, this rate was much lower for other symptoms (Figure 2). Taken together, only 7.1% of patients with fatigue or cognitive disorder received ≥1 BSC measure.

Most measures were pharmacological. For muscle spasticity, baclofen was most frequently prescribed, followed by the combination of cannabidiol/THC (nabiximols) and fampridine, which was also administered for ataxia and fatigue. Fatigue and cognitive impairment were rarely targeted pharmacologically (fampridine: 2.0% of patients; amantadine: 1.5%) and with psychotherapy (1.5%). Compared with other symptoms, muscle spasticity and ataxia were most commonly treated with non-pharmacological therapies, which were dominated by physical/occupational therapy. Moreover, psychotherapy or alternative healing methods were rarely applied (≤2.5% for each symptom).

### Off-label treatments

**Figure 3.**
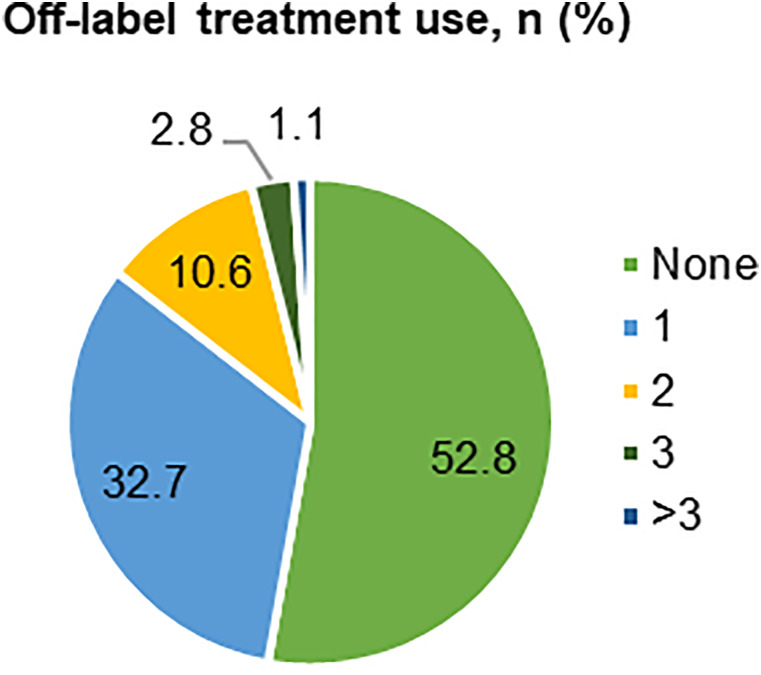
Off-label treatment use. Proportions of patients receiving no or one/multiple off-label treatments with corticosteroids or RRMS-approved DMTs. Percentages are based on the total number of patients (N = 462). RRMS, relapsing-remitting multiple sclerosis; DMT, disease-modifying therapy.

Almost half of the patients were treated with one or multiple off-label medications (47.2%, Figure 3): corticosteroids (32.9%), interferon beta-1a (6.9%), interferon beta-1b (5.2%) and mitoxantrone (5.2%) were most commonly used.

In patients with EDSS <4, 43.7% of patients received ≥1 off-label therapy vs 60.5% in the subgroup with EDSS ≥4. Among these, 44.6% received corticosteroids, compared with 26.8% of patients with a lower EDSS. A wider spectrum of off-label treatments was associated with higher EDSS scores. And a substantially lower percentage of female than male patients received corticosteroids (29.8% vs 36.8%).

### Medical care visits and devices

Only a third of patients were hospitalized ≤2 years prior to the study start, <1% needed an emergency visit, and <12% were treated at a rehabilitation clinic (Table 2). Healthcare providers consulted during this interval included neurologists (89.4%, with a median of 5 visits per patient, range: 0–50 visits), general practitioners (71.0%, median of 6 visits, 0–70 visits), physiotherapists (69.0%, median of 50 visits, 0–300 visits) and occupational therapists (20.6%, median of 50 visits, 0–150 visits), and 43.9% used ≥1 device/aid (28.6% had walking aids; 16.0% were wheelchair-bound).

**Table 2.** Medical care visits in the last 2 years prior to study start.

Number of Patients, n (%)	No Visit	≥1 Visit	1-3 Visits	>4 Visits
Hospitalization	308 (66.7)	154 (33.3)	118 (25.5)	36 (7.9)
Emergency visit	458 (99.1)	4 (0.9)	4 (0.9)	0
Office visit	425 (92.0)	37 (8.0)	33 (7.1)	4 (0.9)
Nursing home	461 (99.8)	1 (0.2)	1 (0.2)	0
Rehabilitation clinic visit	407 (88.1)	55 (11.9)	55 (11.9)	0

Percentages are based on the total number of patients (N = 462).

### EDSS

**Figure 4.**
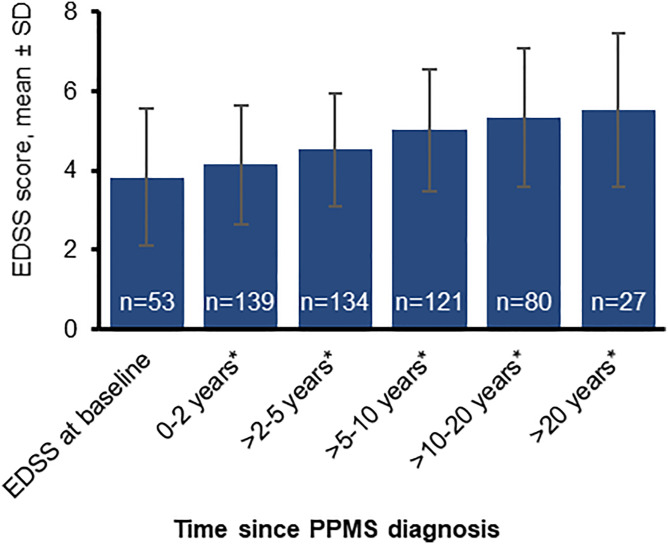
EDSS scores documented at different time intervals following PPMS diagnosis. *After PPMS diagnosis; EDSS at baseline refers to 12 months prior to PPMS diagnosis. EDSS, Expanded Disability Status Scale; PPMS, primary progressive multiple sclerosis; SD, standard deviation.

Continuous disability progression was demonstrated by a mean EDSS score increasing from 3.83 (range: 1.0–8.0; n = 53) at 12 months prior to PPMS diagnosis (baseline) to 5.52 (1.0–8.5; n = 27) at >20 years after diagnosis (Figure 4).

## Discussion

### Study cohort characteristics

RETRO PPMS describes a large cohort of 462 patients with PPMS. Their advanced age and lower female preponderance than in RRMS are in line with other real-world PPMS populations^4,9-13^ and clinical trials.^14,15^ Our study confirms the strikingly long delays between symptom onset and definite diagnosis that were previously reported in other studies with both primary and secondary progressive MS.^13,16,17^

### Symptoms and symptom-specific treatment deficits

The observed high prevalences of various motor impairments, i.e., pyramidal symptoms such as muscle spasticity and gait disturbance, as well as ataxia and bladder dysfunction are known characteristics of PPMS. However, a third of patients were not treated with any physiotherapy/occupational therapy, and only <12% received rehabilitation measures. As expected, more patients with advanced disease (EDSS ≥4) or a longer time since PPMS diagnosis (>10 years) received BSC measures such as physiotherapy or drugs, including off-label treatments. Our data suggest that symptoms such as pain, ataxia, or bladder disorder (52%–71% of affected patients without treatment), remain untreated in many patients. It is also remarkable that although 20% of patients had depression, less than 10% of the affected patients received psychotherapy. This highlights the need for specialized neuropsychiatric care, including psychotherapists,^18^ which would be especially useful to address issues regarding disease-coping. A recent German MS registry analysis found that, despite improved rates for the symptomatic MS treatment of spasticity, pain, ataxia and depression (14%–30% still untreated), the rates of patients untreated for rectal disorders (53%) and bladder dysfunction (47%) remain far too high.^19^

In our study, the prevalence of fatigue was notably lower than anticipated (30.7% of patients), since fatigue was previously reported as one of the most common PPMS symptoms – with rates ranging between 55% in the German MS registry^20^ and 86%^21^ to 95%,^11^ when specific testing for fatigue (e.g. the Fatigue Scale for Motor and Cognitive Functions, FSMC) was applied. The low rate of fatigue documented in this study is likely due to fatigue scales being little known and rarely applied in standard clinical care, which highlights the importance of specific testing.

Comparably, cognitive impairment, which was observed in only 19.5% of patients, has certainly been underreported due to the lack of standardized and appropriate cognitive testing. A real-world PPMS cohort from the NeuroTransData network of German neurology practices similarly reported that only 21% of patients with PPMS (N = 1166) were affected by cognitive impairment (using non-specific and inappropriate dementia screening tests like Demtect or Mini-Mental Status Test, MMST).^11^ Registry-based analyses reported somewhat higher rates (31%, N = 996;^20^ and 27%, N = 2289),^4^ however, these results were solely based on symptom reporting. On the other hand, Ruano et al. formally tested 40 patients with PPMS for cognitive impairment using the sensitive Rao's Brief Repeatable Battery and Stroop test and found that 91.3% of patients with PPMS were affected, and with a higher degree of severity than patients with relapsing MS.^22^ These data underline the critical importance of systematic testing using suitable, standardized and MS-sensitive tools to identify the presence and severity of fatigue (FSMC; Würzburg Fatigue Inventory in Multiple Sclerosis, WEIMuS) and cognitive impairment (Symbol Digit Modalities Test, SDMT; Brief International Cognitive Assessment for MS, BICAMS) in PPMS.

Regarding these “less visible” symptoms, only 7% of the patients affected by fatigue or cognitive impairment received a respective treatment. With the main therapeutic focus being specific motor symptoms, these symptoms are difficult to assess and their treatment is further hampered by limited and complex treatment options. According to data from the German MS registry and the NeuroTransData network, 65% and 81% of patients remained untreated for fatigue and 73% and <70% for cognitive symptoms, respectively.^4,11^ Thus, our data confirm that non-motor and non-focal MS symptoms frequently remain undiagnosed and insufficiently treated,^20^ despite severe impacts on patient quality of life and social participation.

### Treatment with off-label therapies

Ocrelizumab is efficacious^14^ and currently the only approved DMT for PPMS, indicated for the treatment of adult patients with early PPMS characterized by disease duration, level of disability, and imaging features characteristic of inflammatory activity.^5^ Recent real-world data from the ongoing CONFIDENCE study confirmed the safety and effectiveness of ocrelizumab in a PPMS population older than that of other pivotal trials (mean age >50 years) and including patients with comorbidities.^23-25^

It is remarkable that, prior to the availability of ocrelizumab, almost half of all patients with PPMS and over 60% with an EDSS ≥4 received one or more off-label therapies, most commonly corticosteroids or RRMS-approved DMTs. Although no evidence for any substantial effect of these DMTs on short to medium-term disability outcomes in PPMS could be derived from clinical trials^26-28^ – which was also confirmed by an international MSBase study^29^ – prescribing off-label DMTs for PPMS treatment was obviously not an uncommon practice.^9,30^ While this reflects the ongoing medical need for effective DMTs for PPMS, it also may point to a lack of awareness for an optimal, comprehensive treatment: Many neurologists and general practitioners apparently use DMTs that are only efficacious in RRMS as a substitute rather than focusing on adequate long-term symptomatic treatments. This is supported by a German health insurance data-based analysis that calculated the PPMS-associated annual drug treatment costs per patient for the year 2015 at €4,689, approximately 80% of which was for immunomodulatory treatments (prescribed to 22.4% of patients).^12^ Similarly, in a Swedish population-based study, costs for DMTs varied between 40%–70% of total drug expenses over 7 years after PPMS diagnosis (2006–2013).^16^ An Italian study found that PPMS caused average annual healthcare costs of €3,783 per affected person (2015), only 23% of which were spent on drugs.^31^

### Structural deficits and lack of awareness

Müller et al. estimated German healthcare costs for the year 2015 at €13,897 per person for PPMS (>65% non-medication expenses [NME]), €826 symptomatic drug therapy costs [SDT]) and €18,866 for RRMS (<30% NME, €259 SDT), respectively.^12^ While some patients may benefit from high-quality specialist care, there are still considerable treatment deficits. Especially in patients with advanced PPMS, comorbidities and advanced age, not all eligible patients seem to be offered ocrelizumab, and even symptomatic therapies sometimes appear to be abandoned prematurely. These treatment deficiencies may be resultant of severely affected patients often being managed by non-specialist general practitioners and nursing home physicians.^32^

Undoubtedly, there is still a significant lack of comprehensive secondary care infrastructure and resources for outpatient neurological healthcare, with too few patients being referred to specialized centers,^32,33^ even in high-quality, comprehensive healthcare systems such as those in Germany. The German IGES (Institut für Gesundheits- und Sozialforschung) has already criticized severe deficits in symptomatic MS therapy in 2016, and has demanded more comprehensive and structured care concepts involving interdisciplinary networks, including neurologists, neuropsychologists, urologists, radiologists, as well as physical, occupational and speech therapists.^18^

In addition to these problems – which may be representative of the healthcare situation in many European countries – it is important to increase the awareness of primary and secondary care physicians regarding the broad spectrum of PPMS symptoms and available treatment options, including both multidisciplinary symptomatic therapies and approved medications such as ocrelizumab.

### Limitations

Despite some limitations due to retrospective patient chart analysis, such as missing data and recall bias, this study provides valuable insights and systematic data on BSC measures for PPMS in Germany. According to the study design, the analysis used descriptive statistics and focused on absolute and relative frequencies, which may limit understanding of underlying statistically confirmed patterns between different subgroups. The CAP had to be limited to approximately half of the initial target sample size. Underreporting of PPMS symptoms is possible since some symptoms were reported as chronic comorbidities only. Furthermore, less tangible symptoms, e.g. fatigue and cognitive impairment, may be underrepresented in the patient charts. Those symptoms were not systematically diagnosed using normative testing, as suitable test instruments are rarely applied in clinical routine. BSC measures may have also been overlooked as in some cases medications to treat PPMS symptoms may have been reported as concomitant medication only. Moreover, EDSS rating was limited by missing data in a substantial part of patients.

## Conclusion

This thorough retrospective patient chart review illustrates the high disease burden experienced by patients with PPMS and uncovers severe treatment deficits. Many patients appear to receive inadequate BSC measures to treat their symptoms. Particularly fatigue and cognitive impairment are often underdiagnosed, due to neglect or applying the wrong test instruments, and consequently remain undertreated. In conclusion, there is still a widespread need for innovative multidisciplinary care concepts, improvements in neurological care infrastructure and increased awareness for optimal treatment of PPMS.

## Data Availability

Raw data were generated at Roche Pharma AG, Grenzach-Wyhlen, Germany. The data are not publicly available due to restrictions regarding data protection, as they include information that could compromise the privacy of research participants. Anonymized data supporting the findings of this study are available on request from grenzach.biometrics-hub@roche.com.
